# Evidence that inositol 1,4,5-trisphosphate 3-kinase and inositol 1,3,4,5-tetrakisphosphate are negative regulators of platelet function

**DOI:** 10.1016/j.rpth.2024.102326

**Published:** 2024-01-26

**Authors:** Kalwant S. Authi, Sabeeya Khan, Jonathan M. Gibbins, Susan D. Brain

**Affiliations:** 1 School of Cardiovascular and Metabolic Medicine and Sciences, BHF Centre for Research Excellence, London, UK; 2 Institute for Cardiovascular and Metabolic Research, School of Biological Sciences, University of Reading, Reading, UK

**Keywords:** inositol 1,3,4,5-tetrakisphosphate, inositol 1,4,5-trisphosphate 3-kinase, platelet activation, phosphoinositide 3-kinase, phosphatidylinositol 3,4,5-trisphosphate

## Abstract

**Background:**

Inositol 1,3,4,5-tetrakisphosphate (IP_4_) is formed from inositol 1,4,5-trisphosphate (IP_3_) by IP_3_ 3-kinase (ITPK) in most cells. Its function is unknown but has been suggested to be involved in Ca^2+^ entry, IP_3_ regulation, and phosphoinositide 3-kinase antagonism.

**Objectives:**

To better elucidate a function for IP_4_, we tested a specific inhibitor of ITPK (GNF362) on platelets, the effects of IP_4_ directly in permeabilized platelets and its effect on phosphatidylinositol 3,4,5-trisphosphate (PIP_3_) binding to pleckstrin-homology (PH) domain–containing proteins in platelets.

**Methods:**

Human platelets were utilized in whole blood for thrombus formation, in platelet-rich plasma and washed suspensions for aggregation, and for Ca^2+^ studies, or resuspended in high K^+^ and low Na^+^ buffers for permeabilization experiments. Phosphorylation of AKT-Ser^473^ and Rap1-GTP formation were measured by Western blotting and PIP_3_ binding using PIP_3_ beads.

**Results:**

GNF362-enhanced platelet aggregation stimulated by low concentrations of ADP, collagen, thrombin, U46619, and thrombus formation in collagen-coated capillaries. GNF362 induced a transient elevation of Ca^2+^ concentration, elevated basal levels of IP_3_, and enhanced the peak height of Ca^2+^ elevated by agonists. In permeabilized platelets, IP_4_ inhibited GTPγS induced formation of AKT-Ser^473^ phosphorylation and platelet aggregation. IP_4_ reduced GTPγS-stimulated Rap1-GTP levels and potently reduced extraction of RASA3 and BTK by PIP_3_ beads.

**Conclusion:**

ITPK and IP_4_ are negative regulators of platelet function. IP_4_ regulation of PH domain–containing proteins may represent a pathway by which platelet activation may be controlled during thrombosis.

## Essentials

• The function of inositol 1,3,4,5-tetrakisphosphate (IP_4_) in platelets is not known.
• An inhibitor of inositol 1,4,5-trisphosphate 3-kinase (ITPK), GNF362, and IP_4_ were tested.
• GNF362 increased platelet activation and IP_4_ inhibited platelet responses in permeabilized cells.
• ITPK and IP_4_ are negative regulators of platelet activation.

## 1 Introduction

Platelet function is very important in many cardiovascular functions and pathologies such as hemostasis, thrombosis, inflammation, and in the progression of cancer. Current antiplatelet therapy has proven benefit in reducing adverse cardiovascular events but can also lead to increased bleeding. There is thus a need for therapy that is effective but also has fewer side effects. There is increasing laboratory evidence that targeting the phosphoinositide pathways that are linked to increased intracellular Ca^2+^ can inhibit experimental thrombosis and has minor effects on hemostasis [[1], [2], [3], [4], [5]].

Intracellular Ca^2+^ increase is an essential part of platelet activation and occurs when G-protein–linked receptor agonists (such as thrombin, thromboxane A_2_, and ADP) and adhesion receptor agonists (such as collagen and CLEC2) stimulate phospholipase C (PLC) to convert phosphatidylinositol 4,5-bisphosphate to produce inositol 1,4,5-trisphosphate (IP_3_) and 1,2-diacylglycerol (DAG). IP_3_ releases Ca^2+^ from intracellular stores via the IP_3_ receptors (IP_3_R) that then leads to Ca^2+^ entry via store-operated Ca^2+^ entry, and DAG stimulates protein kinase C [6]. IP_3_ is converted to inositol 1,3,4,5-tetrakisphosphate (IP_4_) by the action of IP_3_ 3-kinase (ITPK) or degraded to inositol 1,4-diphosphate (IP_2_) by IP_3_ 5-phosphatase. IP_4_ can be broken down to inositol 1,3,4-trisphosphate and eventually to inositol or it can serve as a precursor for the synthesis of higher inositol phosphates such as IP_5_ and IP_6_ [[7], [8], [9]]. Platelet activation also leads to the generation of phosphatidylinositol 3,4,5-trisphosphate (PIP_3_) from phosphatidylinositol 4,5-bisphosphate by the activation of phosphoinositide 3-kinase (PI3K). Newly generated PIP_3_ acts to recruit signaling proteins that have pleckstrin-homology (PH) domains to the membrane where their manipulation leads to the stimulation of downstream effectors such as protein kinase B (PKB—also known as AKT), Bruton’s tyrosine kinase (BTK), the Ras- and Rap GTP–activating protein RASA3, and many others [10]. In platelet biology, it is generally accepted that sustained activation resulting in aggregation, secretion of granule contents, and procoagulant expression requires all the activation pathways to be maximally operative in a complex and highly coordinated manner. The fine regulation of these pathways is still under investigation and may lead to the development of newer targets for therapeutic development.

The conversion of IP_3_ to IP_4_ and possible functions of IP_4_ have attracted considerable investigations and debate. As the formation of IP_4_ requires ATP and the ITPK enzymes are Ca^2+^-stimulated [9,11], many investigators sought specific functions for IP_4_. Initially, a role for IP_4_ in Ca^2+^ entry was postulated [12], reported to open Ca^2+^ entry channels in endothelial cells [13], to enhance IP_3_-promoted Ca^2+^-activated K^+^ currents [14] and inhibition of IP_3_ 5-phosphatase [15], and to promote IP_3_-dependent Ca^2+^ release from IP_3_R present close to or in the plasma membrane [16]. However, in contrast, IP_4_ has also been suggested to inhibit Ca^2+^ entry channels [17]. Since the formation of IP_4_ from IP_3_ would lead to a decrease in IP_3_ levels, this may in turn limit IP_3_-induced Ca^2+^ release from stores. Structurally, IP_4_ has the same head group as PIP_3_ and is able to potently bind to PH domains such as that found in Bruton’s tyrosine kinase (BTK) [18] and the protein RASA3 [19] (originally called GAP1-^IP4BP^). However as PIP_3_ contains the diacylglycerol moiety, it remains in the plasma membrane, whereas IP_4_ is soluble and thus may function in the cytosolic phase where the majority of these proteins reside. IP_4_ may thus either co-activate or oppose PIP_3_ signaling. In natural killer cells, IP_4_ has been reported to limit interferon gamma secretion and granule exocytosis in part by inhibiting PIP_3_-dependent AKT activation [20]. Similarly, in hematopoietic stem cells, the absence of ITPKB isoform upregulated activation of these cells via the PI3K pathway but impaired their longevity [21]. This suggests that IP_4_ may act to regulate the PI-3K pathway and, in its absence, PIP_3_-dependent activation would be enhanced.

In platelets, there have been limited studies exploring IP_4_ function. O’Rourke et al. reported that IP_4_ mobilizes Ca^2+^ from isolated platelet membranes loaded with Ca^2+^ oxalate [22]. In highly purified platelet plasma membranes, this group has shown that IP_4_ may mobilize Ca^2+^ through a distinct mechanism from that mediated by IP_3_ [23]. Furthermore, cell-permeable PIP_3_ analogs such as DiC8-PIP_3_ induced transient Ca^2+^ elevation in washed platelets [24] and there is evidence that PIP_3_ may bind to the nonspecific Ca^2+^ entry channel TRPC6 [25]. Thus far, no studies have examined any interaction of IP_4_ with the PI3K-AKT pathway in platelets. In light of the potential opposing and controversial roles postulated for IP_4_, this study has re-examined functions for IP_4_ via 2 approaches. We describe the effect of GNF362 [26], a recently described inhibitor of ITPK, on platelet aggregation; on Ca^2+^ mobilization in isolated platelets; and on thrombus formation in collagen-coated capillaries. We examined in a permeabilized platelet model the effects of introducing IP_4_ into the cytosol on aggregation mediated by the nonhydrolyzable analog of GTP (GTPγS), on AKT phosphorylation, Rap1-GTP formation, and in lysates, the binding of PIP_3_ on beads to the PH domain–containing proteins from platelets. Our results suggest that inhibiting ITPK by GNF362 results in the enhancement of platelet activation by the main platelet agonists and increases thrombus formation in collagen-coated capillaries, and in permeabilized cell systems, IP_4_ inhibits AKT phosphorylation, Rap1-GTP formation, and platelet aggregation and displaces PIP_3_ from PH domain–containing proteins such as RASA3 and BTK. Our studies suggest important negative regulatory roles for ITPK and IP_4_ in platelet function.

## 2 Methods

### 2.1 Reagents

DiC8-PIP_3_, In(1,3,4,5)P_4_, In(1,4,5)P_3_ (IP_3_), and GNF362 were obtained from Cambridge Bioscience Ltd. Fura2-AM was obtained from Thermo Fisher Scientific, and U46619 was obtained from Tocris. The phospho-AKT antibody recognizing phospho-Ser^473^ (clone 11E61) was obtained from Upstate. The pan-AKT antibody (C67E7) recognizing Akt1, Akt2, and Akt3 proteins was obtained from Cell Signaling Technology. Goat antimouse antibody linked to horse-radish peroxide (HRP) and Goat antirabbit-HRP were purchased from Millipore. Antibodies to GAP1(IP4BP/RASA3), ITPKB, ITPKA, and BTK were purchased from (Santa Cruz Biotechnology Inc). The anti-Rap1(A+B) and IP_3_-ELISA kit was purchased from Abcam Ltd. GTPγS and RalGDS-RBD beads were obtained from Abcam Ltd. PI(3,4,5)P_3_ PIP beads (PIP_3_ beads) were obtained from Echelon Biosciences (via 2B Scientific). Vena8 Cellix microfluidic chip channels were obtained from Cellix Ltd.

### 2.2 Preparation of human platelets for studies in platelet-rich plasma or washed cells with Fura-2AM loading

Blood was taken from human donors after written consent (with approval from King’s College London and University of Reading Research Ethics Committees) into 0.1 volume of 3.2 % trisodium citrate. Platelet-rich plasma (PRP) was isolated after centrifugation of the blood at 200× *g* for 20 minutes. In studies measuring intracellular Ca^2+^ levels, the platelets were labeled with Fura-2AM in PRP at 37 °C with 3 μM Fura-2AM for 1 hour in the dark. The PRP was then cooled to room temperature and acidified to pH 6.5 with 0.3 M citric acid, and EDTA was added to a final concentration of 3 mM and then centrifuged at 1200× *g* for 20 minutes. The platelet pellet was washed in an EDTA-citrate washing buffer comprising 36 mM citric acid, 1 mM EDTA, 5 mM glucose, 5 mM KCl, 103 mM NaCl (pH: 6.5), and 100 nM PGE_1_. After centrifugation, the platelet pellet was resuspended at 1 to 1.5 × 10^8^ cells/mL in a Hepes Tyrode buffer comprising 10 mM Hepes, 140 mM NaCl, 5 mM KCl, 1 mM MgCl, 5 mM glucose, 0.42 mM NaH_2_PO_4_, and 12 mM NaHCO_3_ (pH: 7.35). For Ca^2+^ studies, 2 mL suspensions were used at 37 °C with continuous stirring using a purpose built spectrofluorimeter (Cairns Research Ltd). Continuous fluorescence measurements were made with excitation at 340 and 380 nm and emission at 510 nm. [Ca^2+^]_i_ is reported as the 340 nm/380 nm ratio (R_340/380_), where indicated ratio values were converted to nM Ca^2+^ using the equation of Grynkiewicz et al. [27] as described by Sage [28].

### 2.3 Preparation of platelets for aggregation, saponin permeabilization, phospho-AKT-Ser^473^ analysis, and Rap1-GTP determination

Washed platelets were isolated as described above (without Fura2 loading) and were resuspended in the Hepes Tyrode’s medium at 2.5 × 10^8^ cells/mL. Incubations were carried out in aggregation cuvettes using a Biodata PAP4 aggregometer using 400-μL suspensions at 37 °C with 1 mM Ca^2+^ and stirring. Reagents were added for the times stated in the Results section and aggregations were measured for 5 minutes. For saponin permeabilization studies, the washed platelets were resuspended in a buffer containing 140 mM KCl, 5 mM glucose, 1 mM MgCl_2_, 0.42 mM NaH_2_PO_4_, 6 mM NaHCO_3_, and 10 mM Hepes made up in HPLC grade water (Sigma-Aldrich) and buffered to pH 7.4. For Rap1 and AKT-phosphorylation studies, the cell count was adjusted to 4 × 10^8^/mL. In typical experiments, 400-μL cells were equilibrated at 37 °C for 3 minutes, and saponin (13 to 15 μg/mL) was added to give an increase of light transmission (aggregation) of between 10% and 20% over an 8 minute period. GTPγS (at either 1 or 2 minutes after saponin addition) or other reagents were added as described in the Figure legends. For Rap1-GTP analysis, the incubations were stopped with ice cold equal volume 2× NP40 lysis buffer containing 100 mM Tris, 400 mM NaCl, 5 mM MgCl_2_, 2% NP40, 20% glycerol, 1% sodium deoxycholate, 2 mM PMSF, 20 μM leupeptin, 0.4 U/mL aprotinin, 2 mM sodium vanadate, and 50 mM NaF (pH: 7.4). Incubations were kept on ice for 20 minutes and then centrifuged at 13,000× *g* for 10 minutes at 4 °C. Then, aliquots of the supernatants were set aside, 2× Laemmli buffer was added, and the they were heated for 5 minutes at 97 °C for total protein inputs and phospho-AKT-Ser^473^ analysis by Western blotting. Remaining supernatants were used for Rap1-GTP pull-down analysis by adding 15-μg RalGDS-RBD beads and incubated for 1 hour with rotation at 4 °C, followed by centrifugation at 14,000× *g* at 4 °C for 20 seconds. The pellets were washed 3 times with 1× NP40 lysis buffer containing inhibitors, finally resuspended in 2× Laemmli buffer, and heated for 5 minutes at 96 °C before Western blotting analysis.

Western blotting was carried out using 10% sodium dodecyl-sulfate–polyacrylamide gel electrophoresis gels, then transferred to polyvinylidene difluoride membranes, and blocked overnight with 5% BSA in TBS-Tween (20 mM Tris [pH: 7.4], 150 mM NaCl, 0.1% Tween). The polyvinylidene difluoride membranes were washed 2 times in TBS-Tween followed by incubation with primary antibody (eg, phospho-AKT-Ser^473^ [at 1/400]) or AKT (pan) antibody at a dilution of 1/1000 in TBS-Tween for 2 hours at room temperature. After 4 washes with TBS-Tween, the membranes were incubated with an appropriate second antibody (eg, goat antimouse [usually 1/5000]) conjugated to HRP in TBS-Tween for 1 hour, followed by 4 washes and detection using chemiluminescence reagents (Thermo Fisher). Images of bands were scanned and analyzed with densitometry using ImageJ. Significance was determined using *t*-test analyzed with GraphPad Prism software. Antibodies used for Western blots in this study with their dilutions included Rap1(A+B) 1/1000; RASA3 1/200, BTK 1/400, ITPKA, and ITPKB 1/200.

IP_3_ levels were estimated using an IP_3_-ELISA kit (Abcam). Platelets were resuspended at 4 × 10^8^ cells/mL, incubated at 37 °C ± 10 μM GNF362 for 1 minute in aggregometer cuvettes with stirring, and stopped with cold 5 mM EDTA/1 μM indomethacin, followed by 3 cycles of freeze–thaw and centrifugation at 13,000× *g* for 10 minutes at 4 °C. Aliquots of the supernatants were analyzed by the IP_3_-ELISA kit according to the manufacturer’s protocol. Experiments involving protein pull down with PIP_3_ beads were carried out as follows. After washing in EDTA-citrate buffer, the platelet pellets were lysed at an equivalent platelet count of 1 × 10^9^ /mL with PIP_3_ lysing buffer comprising 20 mM Hepes (pH: 7.4), 120 mM NaCl, 0.5% NP40, 5 mM EDTA, 5 mM EGTA, 5 mM β-glycerophosphate, 10 mM NaF, 1 mM sodium vanadate, 2 mM PMSF, 20 μM leupeptin, and 0.4 U/mL aprotinin. After 20 minutes at 4 °C, the suspensions were centrifuged at 16,000× *g* for 10 minutes and the supernatants were used for the extraction of PIP_3_ beads. Aliquots (0.4 mL) were incubated with either vehicle (water/dimethyl sulfoxide) or 40 μM DiC8-PIP_3_, or 0.1 to 80 μM IP_4_, and incubated at 4 °C for 30 minutes, followed by addition of either control beads or PIP_3_ attached beads (35 μL equivalent to a calculated concentration of 800 nM PIP_3_) for 90 minutes at 4 °C with rotation. The suspensions were centrifuged at 1000× *g* for 5 minutes at 4 °C in an Eppendorf centrifuge, and the pellets were washed 3 times with lysis buffer and dissolved with 2× Laemmli buffer for Western blotting analysis of the extracted proteins.

Thrombus formation on collagen-coated capillaries was carried out as described previously [29]. Briefly, whole blood was incubated with the fluorescent dye DiOC6 (0.4 μg/mL) for 20 minutes in the dark at 30 °C. Vena8 microfluidic chip channels (with dimensions [width × height × length] of 0.04 × 0.01 × 2.8 cm, respectively) were coated with 2 μL of 0.1-mg/mL collagen for 1 hour at room temperature. Blood was incubated with either vehicle control (dimethyl sulfoxide) or GNF362 (10 μM) for 5 minutes prior to perfusion through the capillaries at a shear rate of 1000 s^−1^ for 5 minutes at 30 °C while placed on the stage of a Nikon A1R laser confocal microscope. The formed thrombi were washed with Tyrode buffer and Z stack images were taken at 3 different areas of the channels. Image J was used to measure the volume of the thrombi.

All statistical analyses were carried out using GraphPad Prism software. Student’s unpaired *t*-test was used to test for statistical significance.

## 3 Results

### 3.1 The ITPK inhibitor GNF362 enhances aggregation of platelets induced by low concentrations of agonists

**Figure 1:**
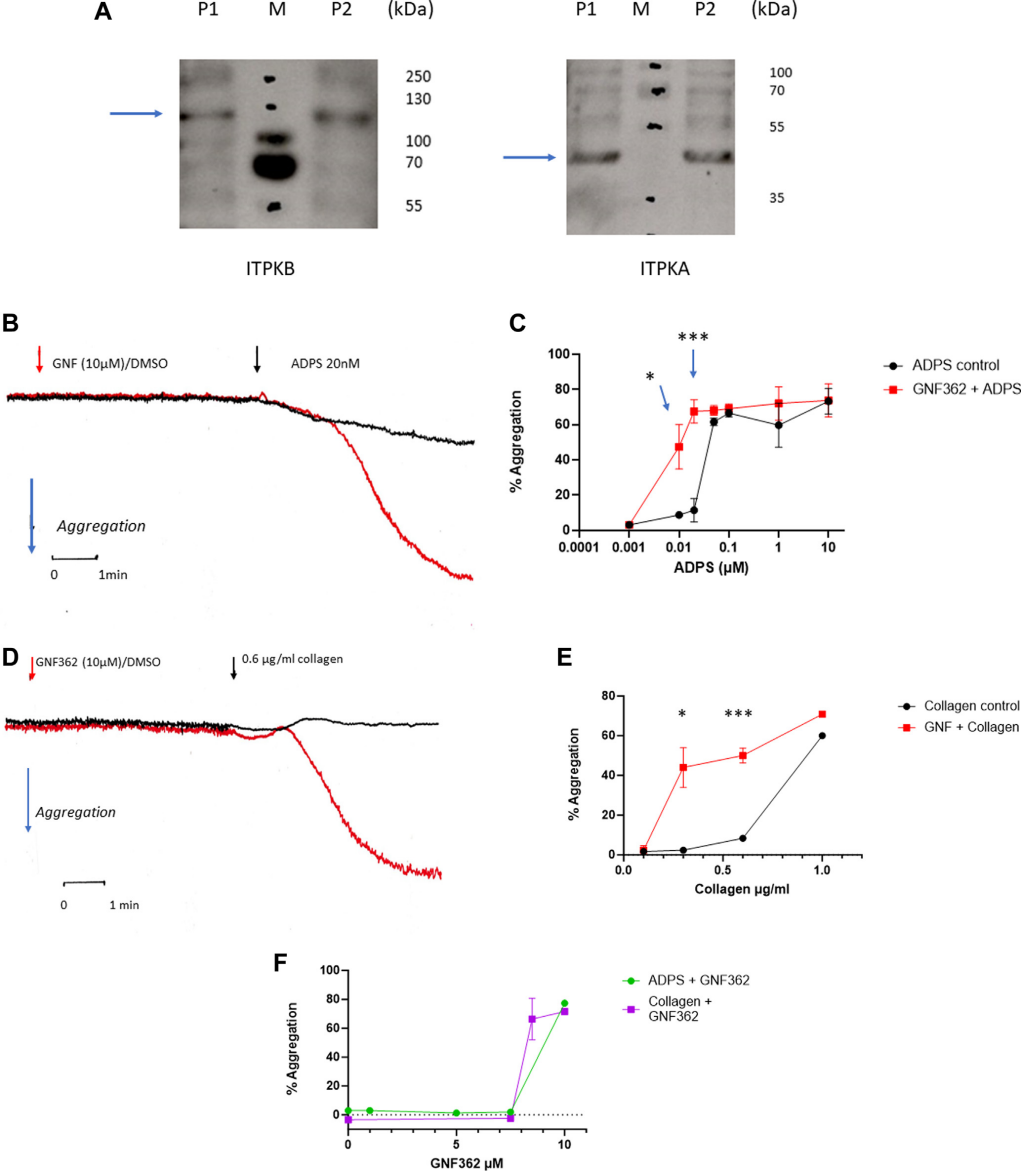
Expression of ITPK isoforms in platelets and effects of GNF362 (GNF). (A) Western blots of platelet lysates P1 and P2 probed with ITPKA and ITPKB antibodies. Arrows point to identified bands for each isoform; M = molecular markers in kDa on the right of each blot. Typical of 4 different platelet preparations. (B, C) GNF362 enhances platelet aggregation to low doses of ADPS and (D, E) to collagen in PRP. (F) Dose–response relationship for GNF362 with ADPS and collagen in PRP. Aggregation traces shown are typical for 3 independent experiments with stirring at 37 °C. Red traces are incubations with GNF362, and black traces are those with vehicle DMSO. GNF362/DMSO was added 5 minutes prior to ADPS or collagen (at indicated concentrations) and recording measured for 5 minutes after agonist addition. Points are means ± SEM (*n* = 3). Student’s unpaired *t*-test; (C) for 10 nM ADPS, *P* = .03, and for 20 nM ADPS, *P* = .0001. (E) For 0.3-μg/mL collagen, *P* = .014; and for 0.6-μg/mL collagen, *P* = .0008. ADPS, 2-methyl-S-ADP; DMSO, dimethyl sulfoxide; ITPK, inositol 1,4,5-trisphosphate 3-kinase; PRP, platelet-rich plasma.

We first tested for the presence of ITPK isoforms expressed in human platelets using Western blotting. Figure 1A shows typical analysis from 2 different platelet preparations (out of 4) with distinct bands at expected sizes of ∼45 kDa and 120 kDa corresponding to ITPKA and ITPKB, respectively, suggesting the presence of both isoforms. Currently, we do not have antibodies to ITPKC to test its presence. We then tested the inhibitor of ITPKs (GNF362) to examine if it affected platelet responses to the major platelet agonists. GNF362 has been shown to inhibit ITPKA, ITPKB, and ITPKC with a IC_50_ of about 20 nM, with total inhibition between 0.2 and 10 μM [17,26]. The effects on ADP-(using 2-methyl-S-ADP [ADPS]) or collagen-induced aggregation were tested using PRP without any further addition of Ca^2+^. Figure 1B, C shows that incubation of PRP with 10 μM GNF362 alone had no noticeable effect on aggregation of platelets measured over a 10-minute period. However, when added 5 minutes prior to addition of low doses of ADPS (10, 20, and 50 nM), there was a marked enhancement of aggregation. The effects were statistically significant, measuring extents of aggregation at 5 minutes induced by 10 and 20 nM ADPS in the presence or absence of 10 μM GNF362 (*P* = .03 and *P* = .0001 for 10 nM and 20 nM ADPS, respectively). At higher concentrations, no enhancement was observed. When collagen-mediated aggregation was studied, the presence of GNF362 again enhanced the aggregation to low concentrations of collagen with no significant effects at higher concentrations (*P* = .014 and *P* = .008 for 0.3 and 0.6 μg/mL collagen, respectively; Figure 1D, E). Noticeably, with collagen, the lag time to shape change was decreased in the presence of GNF362. At 0.3-μg/mL collagen, the lag time to peak of shape change was 2 minutes 5 seconds in the absence and 1 minute 23 seconds in the presence of GNF362 (*P* = .0023), suggesting an enhancement in the rate of activation of this response in the presence of the ITPK inhibitor. With both collagen and ADP in PRP, GNF362 was effective at concentrations of >8 μM, with lower concentrations showing little effect (Figure 1F).

**Figure 2:**
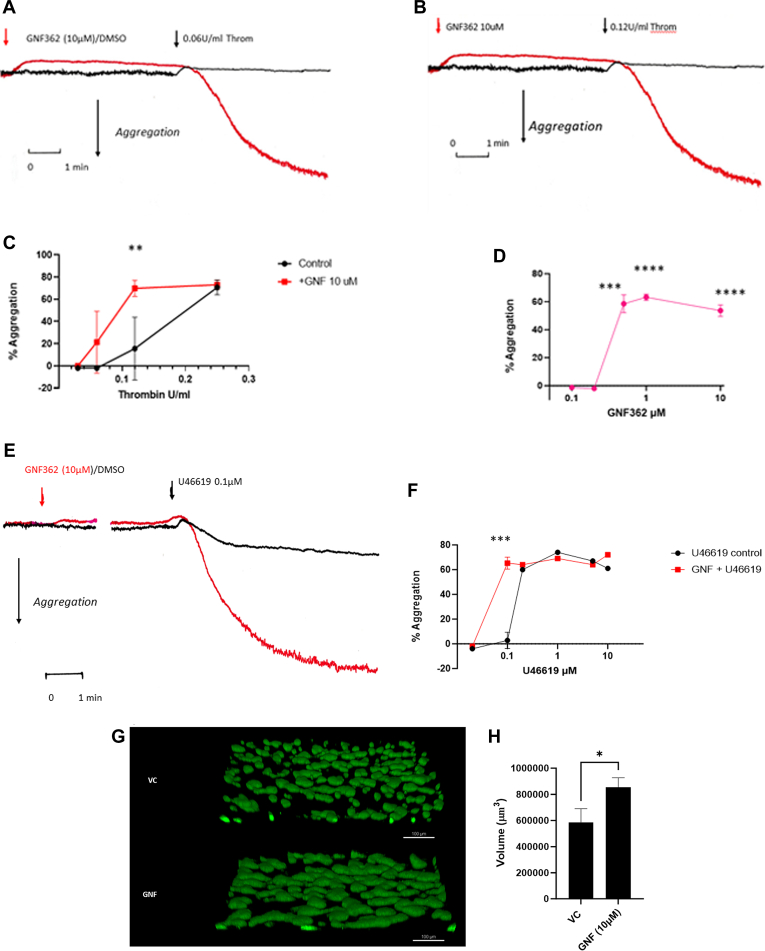
GNF362 enhances aggregation responses to thrombin and U46619 in washed platelets, and thrombus formation in collagen-coated capillaries. (A–F) Washed platelets were incubated in aggregation tubes at 37 °C with stirring. GNF362 (red traces)/DMSO (black traces) was added at indicated times followed by agonist addition after 5 minutes. All incubations contained 1-mM Ca^2+^. Traces are typical of 6 independent experiments. All values are means ± SEM, and *P* was calculated by Student’s unpaired *t*-test; (C) at 0.12-U/mL thrombin, *P* = .0016. (D) Dose–response relationship for GNF362 against thrombin, means ± SEM (*n* = 3); *P* = .004 for 0.5 μM GNF362, *P* = .0001 for 1 μM GNF362, and *P* = .0004 for 10 μM GNF362. (F) At 0.1 μM U46619, *P* = .0001 (*n* = 4). (G, H) Whole blood–induced thrombus formation on collagen-coated capillaries in the presence and absence of 10 μM GNF362. Panel G represents typical confocal images of thrombi formed in collagen-coated capillaries for VC or + GNF362 (GNF); (H) values are means ± SEM (*n* = 3), *P* = .05. DMSO, dimethyl sulfoxide; VC, vehicle control.

Using washed platelets, we tested the effects of GNF362 on thrombin and the thromboxane mimetic U46619. Figure 2A, B shows that addition of 10 μM GNF362 to washed platelets induced a decrease of light transmission, indicating a shape change response. This response was present in the presence or absence of 1 mM extracellular Ca^2+^. Shape change responses were seen by concentrations of GNF362 of >2 μM and within 30 seconds of addition. When tested against thrombin, GNF362 when added 5 minutes prior to agonist addition enhanced the aggregation seen at low concentrations of thrombin (eg, at 0.12-U/mL thrombin, extent of aggregation was 18% ± 12% in the absence and 71.5% ± 2.5% in the presence of 10 μM GNF362; *n* = 6, *P* = .0016 [mean ± SEM]) with no significant difference at concentrations of ≥0.25 U/mL. With washed platelets, GNF362 was effective at ≥0.5 μM (Figure 2D), suggesting that in PRP, it may bind to plasma proteins reducing its potency. Similar effects were seen with U46619 as the agonist. At 0.1 μM U46619, 10 μM GNF362 enhanced the aggregation from 2% ± 3.3% to 65% ± 2.4% (mean ± SEM; *n* = 4, *P* = .0001). At >0.2 μM U46619, there was no difference in the extent of aggregation seen with GNF362.

We have previously shown platelets labeled with the fluorescent dye DiOC6 in whole blood and perfused through collagen-coated capillaries at arterial shear (of 1000 s^−1^) will adhere to the collagen and form thrombi, and this is affected by antibodies to GPIB and STIM1 [29]. We therefore tested if GNF362 would affect the size of thrombi formed in this system. Figure 2G, H shows that 10 μM GNF362 incubated for 5 minutes prior to flow significantly increased the volume of thrombi formed. This confirms that inhibition of ITPK by GNF362 enhances activation by all platelet agonists tested, suggesting that ITPK was an important regulator for both GPCR- and for ITAM-linked receptor agonists and thereby thrombus formation.

**Figure 3:**
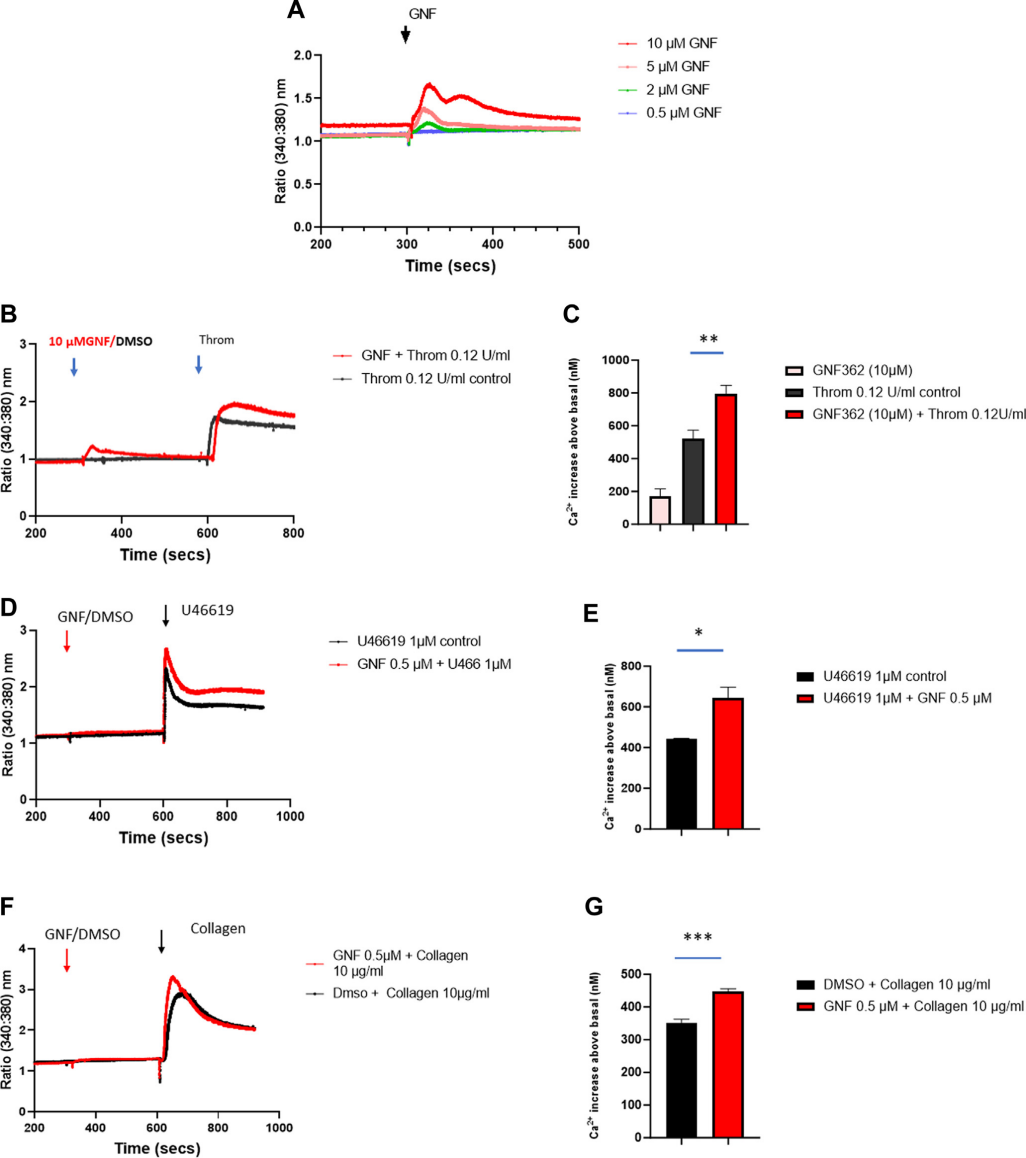
GNF362 enhances Ca^2+^ responses to agonists in Fura-2–labeled platelets. (A) Varying concentrations of GNF362 were added to Fura-2–loaded cells at indicated arrow, and recording representing ratio 340/380 nm excitation was made. Incubations contained 1 mM Ca^2+^. (B) GNF362 (GNF) or DMSO is added at 6 minutes followed by thrombin (0.12 U/mL) at 10 minutes. (C) Statistics for peak increases above basal of Ca^2+^ (nM) for 0.12-U/mL thrombin vs 0.12-U/mL thrombin + 10 μM GNF362 (*P* = .003; values are means ± SEM; *n* = 3); Student’s unpaired *t*-test used for all *P* values. (D) Ca^2+^ responses for 1 μM U46619 vs 1 μM U46619 + 0.5 μM GNF362. (E) Peak increases of Ca^2+^ (nM) for panel D (means ± SEM; *n* = 3, *P* = .022). (F) Ca^2+^ responses for 10-μg/mL collagen vs 10-μg/mL collagen + 0.5 μM GNF362. (G) Peak increases of Ca^2+^ (nM) for panel F (means ± SEM; *n* = 3, *P* = .0003). DMSO, dimethyl sulfoxide.

We then tested if the presence of GNF362 would lead to changes in Ca^2+^ levels in Fura-2–labeled platelets. Figure 3A shows typical responses following addition of 1- to 10-μM GNF362 to Fura-2–labeled cells in the presence of 1 mM Ca^2+^ causing transient elevation of Ca^2+^ that peaked within 30 seconds and then returned to near basal levels within 5 minutes (maximal response seen between different platelet preparations ranged between 150 and nearly 400 nM but was transient and returned to near basal levels by 5 minutes). Ca^2+^ elevation by GNF362 correlated with the shape change response seen in washed platelets. Ca^2+^ elevation by GNF362 was also observed in platelets incubated with 0.1 mM EGTA to chelate extracellular Ca^2+^ and thus indicates an initial Ca^2+^ release from stores (results not shown). Again, this was transient and went back to near basal levels within 5 minutes. We tested for effects on agonist-induced Ca^2+^ elevation after 5-minute incubation of GNF362. The presence of 10 μM GNF362 significantly enhanced the peak height of Ca^2+^ elevation seen with 0.12-U/mL thrombin (Figure 3B), with statistics shown as peak increase (nM Ca^2+^) above basal levels (Figure 3C; *P* = .003). When GNF362 was tested at a concentration of 0.5 μM that did not cause Ca^2+^ elevation by itself, there was still an enhancement of the Ca^2+^ response to agonists. Typically, Figure 3D–G shows that 0.5 μM GNF362 enhanced the peak responses seen with 1 μM U46619 and 10-μg/mL collagen. To test if the presence of GNF362 would affect IP_3_ levels, we carried out preliminary studies measuring IP_3_ levels using an IP_3_-ELISA kit as described in the Methods. Platelets resuspended at 4 × 10^8^ cells/mL and incubated at 37 °C with 10 μM GNF362 for 1 minute increased basal levels of IP_3_ from 151.7 ± 10.9 pg/mL to 193.0 ± 10.3 pg/mL (*n* = 3; *P* = .04). This suggests that inhibition of ITPK by GNF362 may increase basal levels of IP_3_ contributing to the activation seen.

### 3.2 IP_4_ inhibits activation of saponin-permeabilized platelets

**Figure 4:**
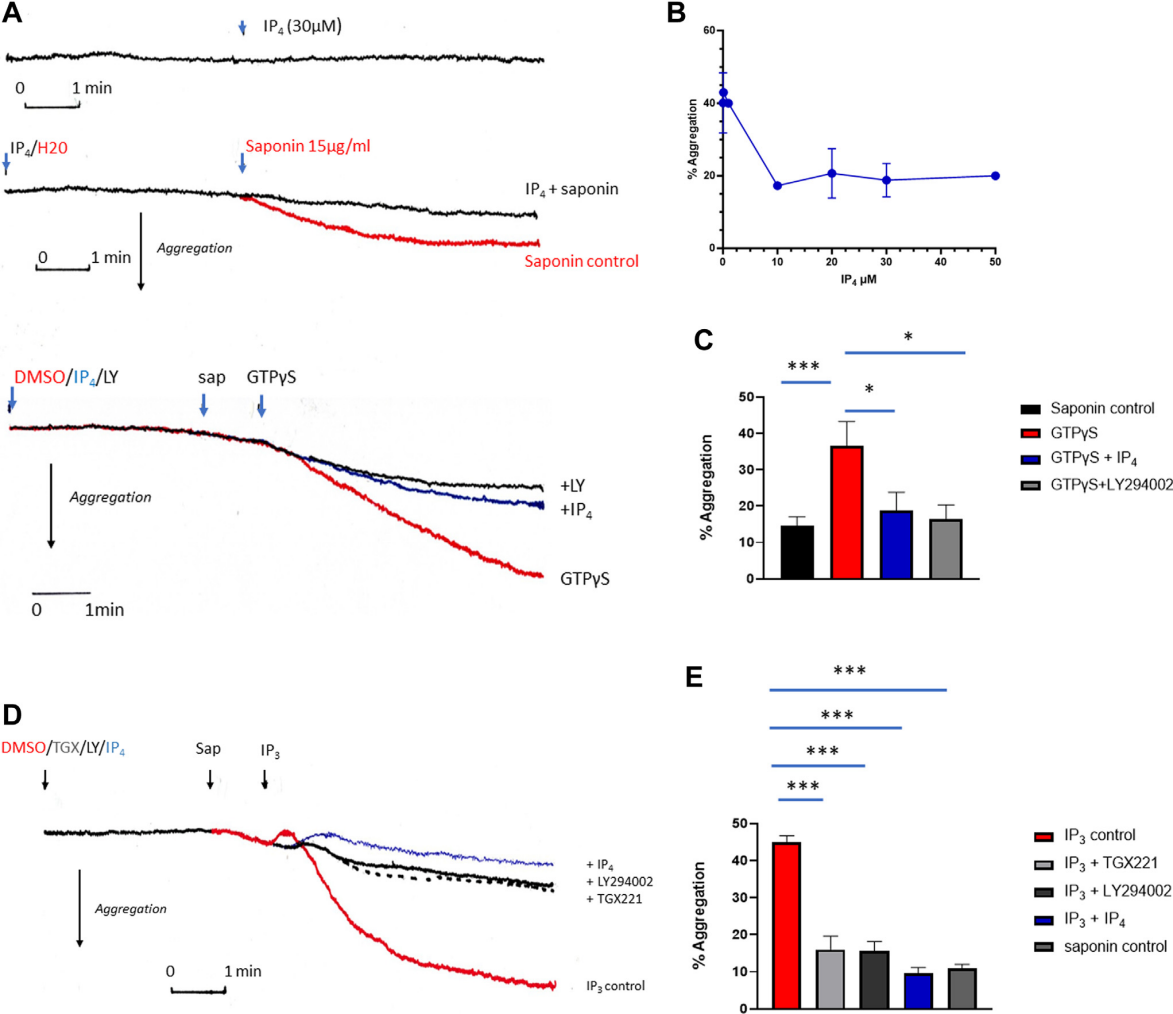
IP_4_ inhibits aggregation of saponin-permeabilized platelets. Platelets were resuspended in high K^+^ medium (see Methods for details) and placed in aggregation cuvettes at 37 °C. After 3 minutes, equilibration reagents were added as indicated. (A) Top part: IP_4_ (30 μM) alone; middle part: saponin (15 μg/mL, red trace) in the presence and absence of IP_4_ (30 μM); and bottom part: saponin (sap; 15 μg/mL) + GTPγS (100 μM, red trace) in the presence of LY294002 (LY; 25 μM, black trace) or IP_4_ (30 μM, blue trace). Saponin control left out for clarity. (B) Dose–response relationship for IP_4_ inhibition of aggregation induced by GTPγS (means ± SEM; *n* = 3). (C) Effect of IP_4_ (30 μM) and LY294002 (25 μM) on 100 μM GTPγS–induced aggregation of saponin-permeabilized platelets (means ± SEM, *n* = 3). *P* values calculated using Student’s unpaired *t*-test: *P* = .0009 for saponin control vs GTPγS; *P* = .0125 for GTPγS vs GTPγS + IP_4_; and *P* = .016 for GTPγS vs GTPγS + LY294002. (D) Effect of LY294002 (25 μM), TGX221 (10 μM), and IP_4_ (30 μM) on 90 μM IP_3_–induced aggregation of saponin-permeabilized platelets. (E) Statistics of panel D: values are means ± SEM (*n* = 3)—*P* = .0002 for IP_3_ vs IP_3_ + TGX221; *P* = .0001 for IP_3_ vs IP_3_ + LY294002; *P* = .0001 for IP_3_ vs IP_3_ + IP_4_; and *P* = .0001 for IP_3_ vs saponin control. IP_3_, inositol 1,4,5-trisphosphate; IP_4_, inositol 1,3,4,5-tetrakisphosphate.

The product of ITPK (IP_4_) is water-soluble and not able to cross cell membranes. To test if it could exert actions on cell activation, we tested for effects on various activities measurable in saponin-permeabilized platelets. We have previously shown saponin at 13 to 15 μg/mL to permeabilize washed platelets within 1 minute (as determined by release of adenine metabolites) and will cause a small increase in light transmission as measured by aggregometry, representing ∼12% to 16% aggregation over an 8-minute incubation [30]. We have also shown the nonhydrolyzable analog of GTP (GTPγS) and IP_3_ to further accelerate aggregation of saponin-treated platelets and induce thromboxane B_2_ formation and dense granule secretion [31,32]. GTPγS and IP_3_ are ineffective in intact cells, consistent with actions at intracellular sites. Therefore, IP_4_ was tested for effects on its own or if it altered GTPγS- or IP_3_-induced activation. Figure 4A shows that addition of 30 μM IP_4_ (tested range: 1-80 μM) to intact platelets caused no measurable effect on aggregation traces. Incubation of platelets with 14-μg/mL saponin caused an approximately 10% to 15% increase of light transmission (% aggregation) over 8 minutes. The addition of 30 μM IP_4_ either 3 minutes before or 2 minutes after saponin addition had no further effect and mostly reduced the saponin effect by 2% to 3%, suggesting that it was not stimulatory. When tested with GTPγS-induced aggregation, IP_4_ when added 3 minutes before saponin caused an inhibition of the GTPγS-induced aggregation to the level seen with saponin alone (*P* = .0125, *n* = 3). Concentrations of IP_4_ at 0.1 to 1 μM had little effect but those at ≥10 μM showed significant inhibitory effects on aggregation (Figure 4B, C). The PI3K inhibitor LY294002 (tested at 25 μM to inhibit all isoforms) also inhibited GTPγS-induced response, suggesting that PI3K was important in this system. Similar inhibitory effects by IP_4_ were seen when IP_3_ was used to cause aggregation of permeabilized platelets (Figure 4D, E). No additive or synergistic effects between IP_4_ and IP_3_ were observed, suggesting distinct actions. The PI3Kβ isoform specific inhibitor TGX221 was equally effective as LY294002 at inhibiting the IP_3_-mediated aggregation response, suggesting that PI3Kβ was the main isoform activated in this model.

**Figure 5:**
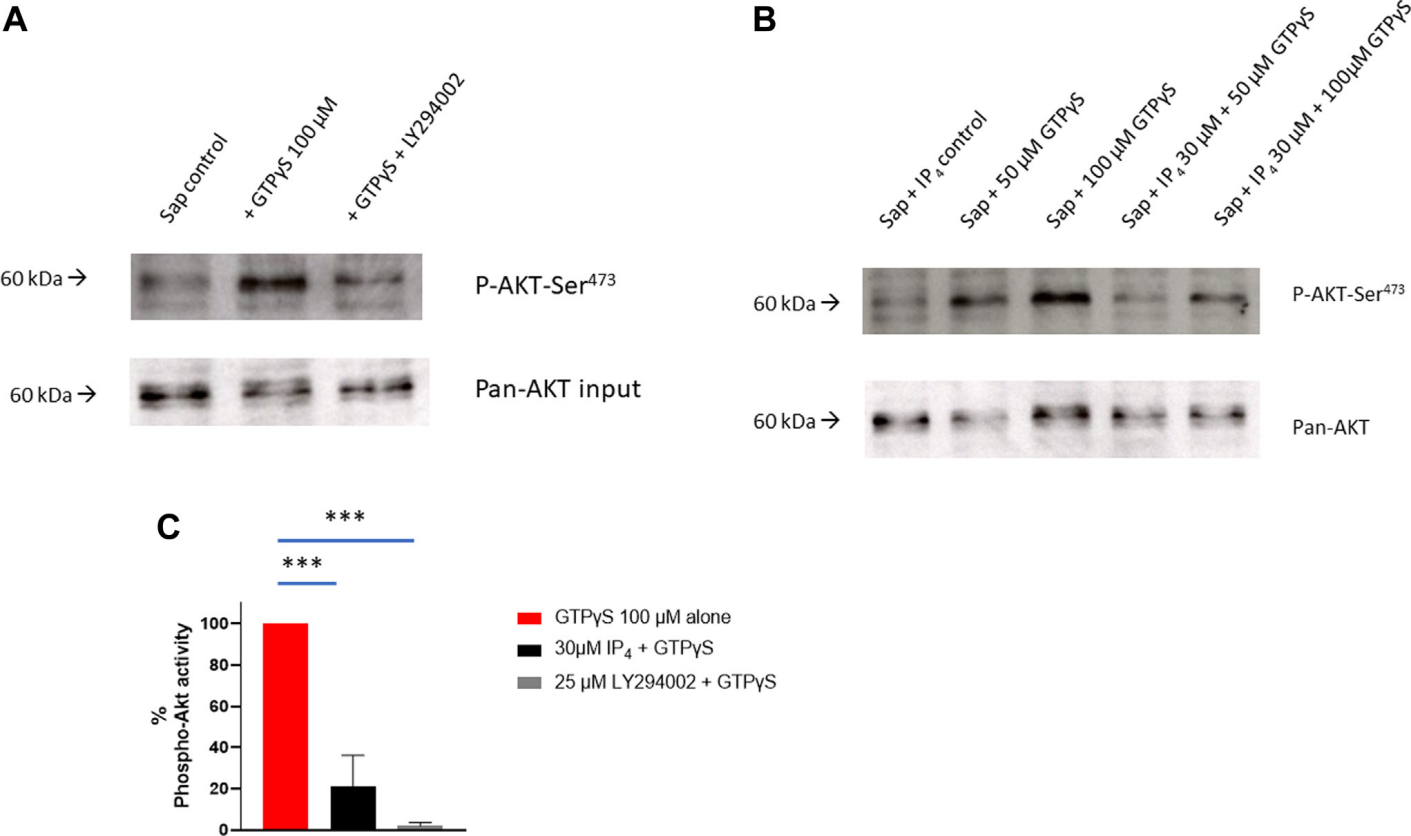
IP_4_ inhibits GTPγS-induced AKT-Ser^473^ phosphorylation in saponin-permeabilized platelets. Experiments were carried out as in Figure 4. At the end of the incubations, cells were lysed with NP40 lysis buffer (see Methods for details). After centrifugation and addition of Laemmli buffer, the lysates were estimated for AKT-Ser^473^ phosphorylation using phospho-AKT-Ser^473^ antibody and total AKT (with pan-AKT antibody, depicting total input) by Western blotting. (A) LY294002 (25 μM) was tested against 100 μM GTPγS. (B) Fifty micromolar and 100 μM concentrations of GTPγS were tested against 30 μM IP_4_. (C) Statistics of 100 μM GTPγS vs 30 μM IP_4_ and 25 μM LY294002 inhibition of AKT-Ser^473^ phosphorylation; values are means ± SEM (*n* = 4 for GTPγS, *n* = 4 for GTPγS + IP_4_, and *n* = 3 for GTPγS + LY294002). ∗∗∗*P* = .0001 calculated using Student’s unpaired *t*-test. IP_4_, inositol 1,3,4,5-tetrakisphosphate.

The inhibitory effect of IP_4_ may be due to actions at multiple intracellular targets. As IP_4_ has the same headgroup as PIP_3_ and both LY294002 and TGX221 were inhibitory, we considered that it may target PI3K-dependent pathways. A commonly used measure of PI3K activity is measurement of AKT phosphorylation [24]. At concentrations that cause aggregation, GTPγS induced the phosphorylation of AKT-Ser^473^ as measured using Western blotting with a specific phospho-AKT-Ser^473^ antibody (Figure 5A–C). GTPγS-stimulated phosphorylation of AKT-Ser^473^ was inhibited by LY294002 (25 μM; *P* = .0001, *n* = 3) and by 30 μM IP_4_ (*P* = .0001, *n* = 4 against 100 μM GTPγS). IP_4_ thus inhibited a PI3K effector activity in permeabilized platelets.

**Figure 6:**
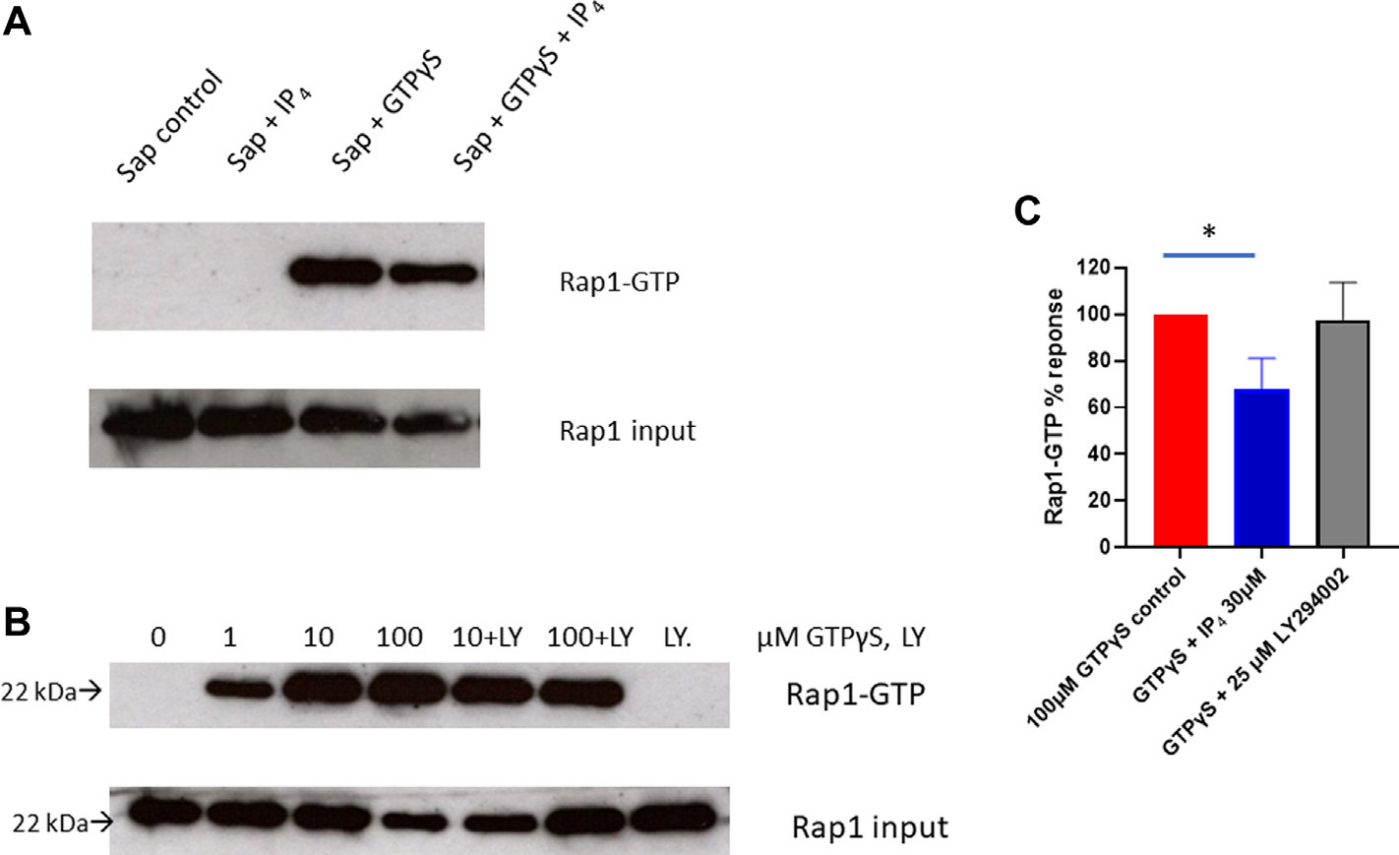
GTPγS stimulates Rap1-GTP formation in saponin-permeabilized platelets and the effect of IP_4_. Experiments were carried out as in Figure 4, Figure 5. Rap1-GTP was extracted from NP40 lysates using RalGDS-RBD beads and Rap1-GTP determined using Western blotting with anti–Rap1(A+B) antibody. Blots shown are typical of 3 experiments. Rap1 input was determined using aliquots of NP40 lysates. (A) Incubations were carried out for 5 minutes with 100 μM GTPγS and 30 μM IP_4_. (B) Varying concentrations of GTPγS in the presence and absence of 25 μM LY294002 for 5 minutes. (C) Statistics of 30 μM IP_4_ or 25 μM LY294002 effects on 100 μM GTPγS–stimulated Rap1-GTP formation. Values are means ± SEM (*n* = 5 for GTPγS, *n* = 3 for GTPγS + IP_4_, and *n* = 3 for GTPγS + LY294002). ∗*P* = .012, Student’s unpaired *t*-test. IP_4_, inositol 1,3,4,5-tetrakisphosphate.

IP_4_ is known to bind the Ras GTPase–activating protein RASA3 [19]. RASA3 is known to inactivate Rap1 by increasing the conversion of Rap1-GTP to Rap1-GDP [33,34]. We therefore examined activation of Rap1 with GTPγS and tested to see if IP_4_ affected the Rap1-GTP status. Figure 6A–C shows that in saponin-permeabilized platelets, GTPγS induces activation of Rap1 as estimated by pull-down assays using RalGDS-RBD beads. When tested for effects with IP_4_ alone, there was no effect on Rap1-GTP levels, but at 30 μM, it caused a 27 % inhibition (*P* = .012) of 100 μM GTPγS–induced formation of Rap1-GTP. Under the same conditions, the PI3K inhibitor LY294002 had no significant effect on 100 μM GTPγS–induced Rap1-GTP formation. This suggested that GTPγS induced Rap1-GTP independently of PI3K activity. IP_4_ may possibly act directly on RASA3, resulting in reduced Rap-1-GTP levels.

### 3.3 IP_4_ inhibits extraction of platelet PH domain proteins using PIP_3_ beads

**Figure 7:**
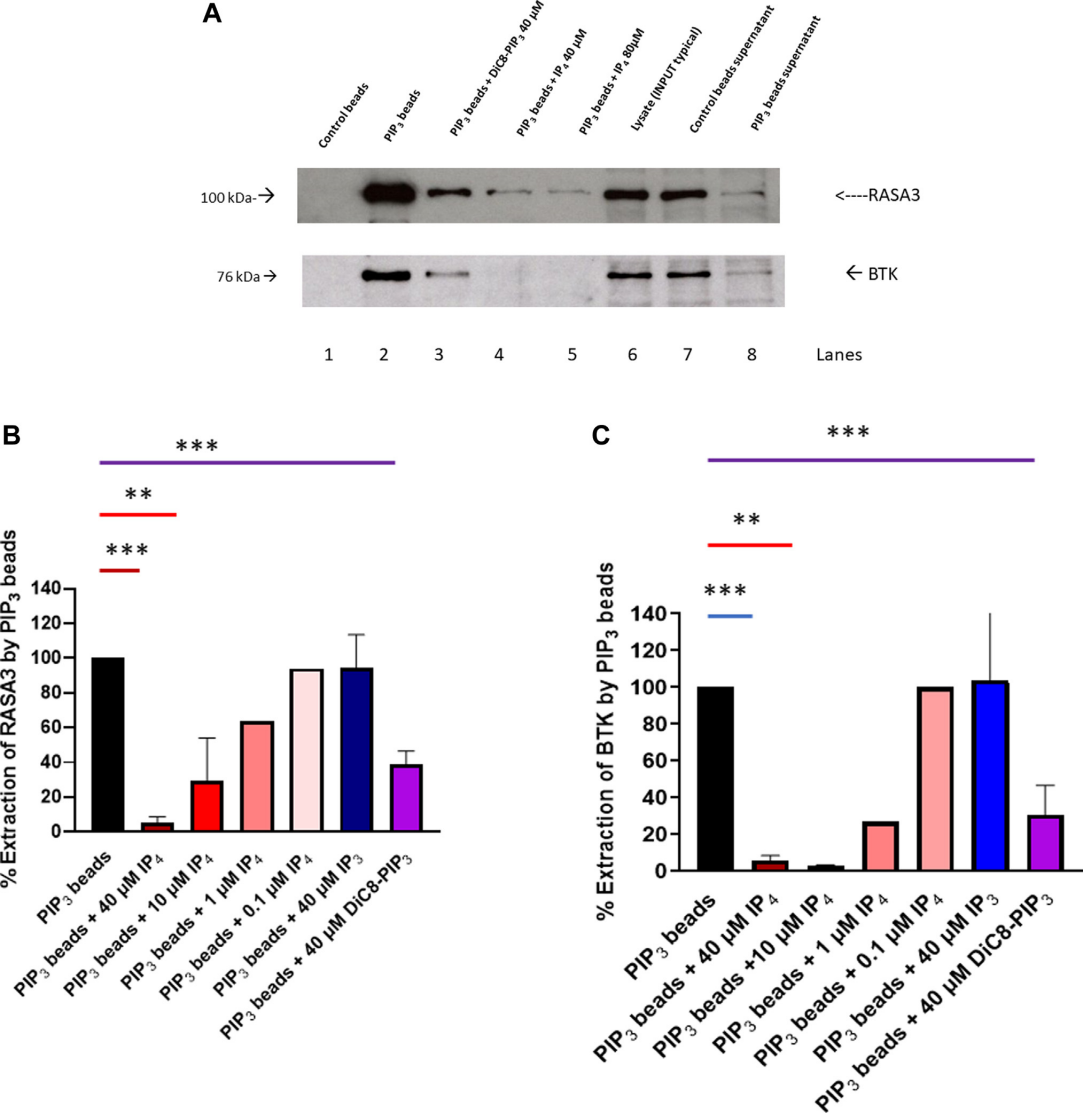
Effect of IP_4_ on extraction of RASA3 and BTK from NP40 platelet lysates by PIP_3_ beads. Resting platelet lysates were incubated with either control beads or PIP_3_ beads with or without exogenous inositol lipids and extracted, and proteins were detected by Western blotting. (A) Top: detection of RASA3; bottom: detection of BTK. Lysates were incubated with control beads (lane 1), PIP_3_ beads (lane 2), PIP_3_ beads + 40 μM DiC8-PIP_3_ (lane 3), PIP_3_ beads + 40 μM IP_4_ (lane 4), PIP_3_ beads + 80 μM IP_4_ (lane 5), lysate (1/40 input of protein for each incubation, lane 6), post control bead lysate supernatant (1/40 of total, lane 7), and post PIP_3_ beads supernatant (1/40 of total, lane 8). Images are typical of 3 different experiments. (B) Effect of varying doses of IP_4_, IP_3_, and DiC8-PIP_3_ on RASA3 extraction with PIP_3_ beads and (C) the same for BTK. Values are means ± SEM (*n* = 4); ∗∗*P* = .0019 and ∗∗∗*P* = .0001 calculated using Student’s unpaired *t*-test. BTK, Bruton’s tyrosine kinase; IP_3_, inositol 1,4,5-trisphosphate; IP_4_, inositol 1,3,4,5-tetrakisphosphate; PIP_3_, phosphatidylinositol 3,4,5-trisphosphate.

In order to understand the mechanism of inhibitory action of IP_4_, we sought to determine if IP_4_ would displace PIP_3_ from PH domains of known PIP_3_ binding proteins. PIP_3_-coupled beads have been used to capture PIP_3_ binding proteins from cellular lysates including platelets [35]. Therefore, platelet lysates were incubated with control or PIP_3_ beads in the presence or absence of DiC8-PIP_3_ or IP_4_. After binding, the beads were isolated and analyzed by Western blotting for the pull-down of PIP_3_-binding proteins. Figure 7A shows that RASA3 is efficiently extracted by PIP_3_ beads and that this extraction was competed for by 40 μM DiC8-PIP_3_ (Figure 7A [lanes 2 and 3]). Examination of the platelet lysate after PIP_3_ bead incubation showed 87% reduction in RASA3 protein compared with control beads (compare lanes 7 and 8), confirming PIP_3_-mediated extraction. The inclusion of IP_4_ potently inhibited extraction of RASA3 by PIP_3_ beads (lanes 4 and 5). Competition for PIP_3_ binding to RASA3 was evident at 1 μM IP_4_, with increasing concentrations showing stronger displacement such that at 40 μM, there was >90% inhibition of RASA3 extraction on PIP_3_ beads (statistics shown in Figure 7B). Under the same conditions, 40 μM IP_3_ was without effect, indicating distinct actions of IP_4_ compared to IP_3_. In a similar manner, IP_4_ also reduced extraction of the PH domain–bearing tyrosine kinase BTK (Figure 7A, C). Compared to IP_4_, exogenously added DiC8-PIP_3_ was marginally less potent at competing with PIP_3_ beads. This may reflect the lack of intact membranes in the lysates with which this reagent will fuse as DiC8-PIP_3_ is highly polarized and/or that IP_4_ has a higher binding affinity. Our findings suggest IP_4_ to strongly antagonize PI3K effectors to inhibit cell activation.

## 4 Discussion

This study identifies ITPK and IP_4_ as negative regulators of platelet activation and represents a mechanism to finetune the activation process induced by all the agonists tested thus far. IP_4_ formation is an ATP-requiring Ca^2+^-dependent pathway to metabolize the Ca^2+^ mobilizing messenger IP_3_ [11,36]. Since its discovery, various investigators have described possible functions for IP_4_, which included roles in Ca^2+^ influx [12,13,23], Ca^2+^ entry into the nucleus [37], cooperation at the level of the IP_3_R [38], and inhibition of the IP_3_-5’-phosphatase [15]. In contrast, negative roles for IP_4_ in Ca^2+^ entry have also been described in immune cells [17,39], including an inhibitory effect for IP_4_ on the Orai1 channel [17] and a regulatory function for IP_4_ in the PI3K pathway in reducing AKT phosphorylation in immune cells [20,21,26,40,41]. Thus, an established role for IP_4_ in cell function is not present. Only recently have specific inhibitors of ITPK been described (such as GNF362), and their use suggest an antagonist role for IP_4_ in PI3K-dependent effects [26,42]. We report here human platelets to express at least ITPKA and ITPKB [43], and the presence of GNF362 resulted in an enhancement of platelet aggregation and Ca^2+^ elevation induced by all agonists tested and increased thrombus formation on collagen-coated capillaries (Figure 1, Figure 2, Figure 3). There appears increased potency of the reagent in washed vs PRP or whole blood, and this may reflect some binding to plasma proteins. In washed platelet suspensions, GNF362 at 5 to 10 μM also induced shape change and a transient increase of intracellular Ca^2+^. This is most likely due to inhibition of basal turnover of IP_3_ to IP_4_, resulting in transiently higher IP_3_ levels leading to Ca^2+^ elevation and activation. This suggests that even though platelets express both ITPKs and IP_3_ 5-phosphatases to metabolize IP_3_ [44], ITPK may be the preferred route for IP_3_ metabolism. Our future studies will examine the kinetics of this pathway in detail.

Our studies on permeabilized platelets show IP_4_ as an antagonist of PI3K effectors. We have previously shown IP_3_ and GTPγS to accelerate aggregation in saponin-permeabilized platelets, which results from the release of Ca^2+^ from intracellular stores, activation of G proteins, and formation of thromboxane A_2_ [[30], [31], [32]]. Here, we report IP_4_ to inhibit AKT phosphorylation and aggregation, and to a modest extent Rap1-GTP formation. GTPγS will activate G proteins by displacing the resting GDP bound to the GTP-bound states. Rap1 in the GTP-bound form is important for Talin-dependent activation of the αIIbβ3 integrin complex (inside-out signaling) that is essential for rapid platelet aggregation [45,46]. The effect of IP_4_ on active Rap-1 was inhibitory, suggesting an action of either directly stimulating RASA3 or displacing any bound PIP_3_ from RASA3, leading to a reduced level of RAP1-GTP. The modest inhibition is probably related to the relative nonhydrolysable nature of GTPγS acting directly on Rap1. GTPγS addition led to stimulation of the PI-3Kβ isoform, as the phosphorylation of AKT was inhibited by both LY294002 and the β-isoform specific inhibitor TGX-221. The IP_4_-induced inhibition of AKT phosphorylation suggests that PI3K effectors are the targets of IP_4_ action. AKT itself is known to be stimulatory in platelet activation [47]; however, how it may be linked to integrins or other aspects of platelet activation still needs to be better understood.

To understand IP_4_ actions further, we examined PIP_3_ binding to RASA3 and BTK, which were both almost totally extracted by PIP_3_ beads [35]. IP_4_ potently displaced RASA3 and BTK from PIP_3_ beads, antagonizing PIP_3_ action. In our hands, extraction of AKT with PIP_3_ beads was faint, which may reflect lower copy numbers in platelets than from the extraction of RASA3 or BTK, or the affinity of the antibody may not be sufficient and therefore accurate analysis was not possible (results not shown). PI3K effectors play important enhancement roles at different stages of signaling. For ITAM-linked receptors, PI3K is important in activating PLCγ2 via BTK [48]. Lack of BTK in human platelets results in decreased collagen-induced tyrosine phosphorylation of PLCγ2, Ca^2+^ mobilization, and aggregation [49]. The inhibition of BTK by IP_4_ corroborates the enhancement of collagen-induced effects in intact platelets by GNF362. Although BTK deficiency did not affect thrombin-induced responses in the study of Quek et al. [49], it is of interest that all PLC isoforms (except the ζ form) utilized by GPCRs have PH domains and therefore a potential for modulation by phosphoinositides and IP_4_ [50]. Further studies are required to examine if IP_4_ affects PLC isoforms important for GPCR-linked receptors. In thymocytes notably, IP_4_ has been reported to increase IL-2–stimulated T-cell kinase (ITK, another Tec kinase) activity that subsequently stimulates PLCγ1 [51]. ITK has not been shown to be present in platelets, so if this reflects a cell-specific activity remains to be clarified. RASA3 is suggested to have a prominent role in inactivating Rap1, and its inhibition by the P2Y_12_R-PI3K–dependent or P2Y_12_R-PI3K–independent pathway is considered central to the sustained activation of Rap1 and stable integrin-dependent aggregation [33,34,52,53]. However, there has been to date no direct demonstration that PIP_3_ inhibits RASA3. With most PI3K effectors, the binding of PIP_3_ would result in activation of downstream targets (eg, with AKT) rather than inhibition (as proposed with RASA3), and therefore, this mechanism requires further elucidation. In intact platelets, exogenous addition of DiC8-PIP_3_ will stimulate Ca^2+^ elevation transiently, increase thromboxane production, and stimulate AKT phosphorylation and aggregation [24]. However, in the saponin permeabilization model, addition of DiC8-PIP_3_ has thus far been ineffective and did not affect GTPγS-stimulated Rap1-GTP formation (results not shown). It has been suggested recently that PI3K stimulated production of PIP_3_ and that its binding to RASA3 may restrict RASA3 to the membrane and limit its ability to inactivate Rap1 [54]. IP_4_, which is generated in the cytosol, will be freely available to act on RASA3 to control Rap1.

**Figure 8:**
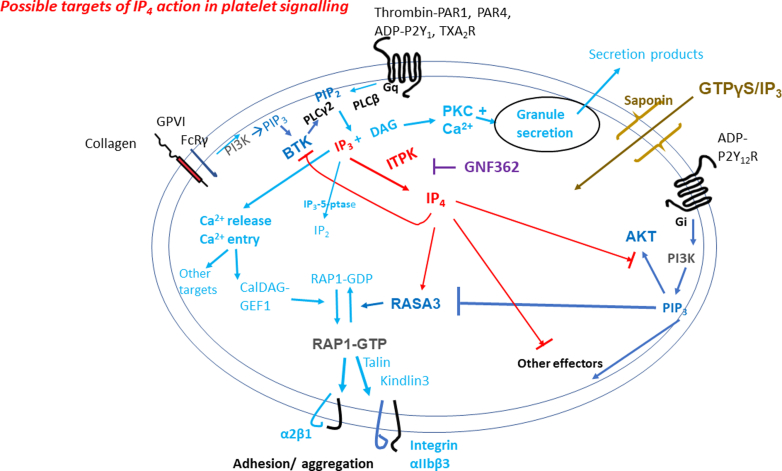
Possible targets of IP_4_ action in platelet signaling. G-protein–linked receptor agonists (such as thrombin, ADP, and thromboxane A_2_) and ITAM-linked agonist (such as collagen) activate PLCβ and PLCγ2, respectively. The resulting IP_3_ and DAG elevate Ca^2+^ and activate PKC. The elevated Ca^2+^ activates many Ca^2+^-driven processes including CalDAG-GEF1 to promote Rap1-GTP formation, which promotes Talin to activate integrins such as αIIbβ3. IP_3_ can be metabolized to IP_4_ by ITPK or to IP_2_ via IP_3_ 5-phosphatase. PI3K can be activated as a consequence of ITAM signaling, stimulating BTK and PLCγ2. Additionally, G-protein–linked receptors, principally ADP-P2Y_12_R, can stimulate PI3K, resulting in activation of effectors such as AKT, and proposed RASA3 inhibition. In saponin-permeabilized platelets, GTPγS/IP_3_ can access the intracellular space and activate G proteins, including Rap1, Ca^2+^ elevation, and PI3K activation. IP_4_ is suggested to antagonize PI3K effectors by binding to and/or displacing PIP_3_ from PH domains. RASA3, BTK, AKT, and other PH domain containing proteins represent potent IP_4_ targets. GNF362 inhibits ITPK, resulting in decreased IP_4_, increased IP_3_ levels, and an enhancement of agonist function. For further details, see Discussion. DAG, 1,2-diacylglycerol; IP_3_, inositol 1,4,5-trisphosphate; IP_4_, inositol 1,3,4,5-tetrakisphosphate; ITPK, inositol 1,4,5-trisphosphate 3-kinase.

In summary, our studies suggest ITPK and IP_4_ to be important negative regulators of platelet activation by controlling levels of IP_3_ and antagonizing PIP_3_ actions. Figure 8 outlines possible mechanisms for IP_4_ with actions at the level of RASA3, BTK, and AKT. We predict many more PH domain proteins to be affected by IP_4_ and further studies are underway to elucidate their importance. The increased synthesis or the development of cell-permeable mimetics of IP_4_ may represent useful strategies for antithrombotic therapeutics.
